# Supplementary material for: Increased warm water intrusions could cause mass loss in East Antarctica during the next 200 years
Source: Nat Commun. 2023 Apr 1;14:1825. doi: 10.1038/s41467-023-37553-2 (PMC10067810; doi:10.1038/s41467-023-37553-2)
Supplement: Supplementary file 1 — Supplementary Information [file 41467_2023_37553_MOESM1_ESM.pdf]

Increased warm water intrusions could cause mass loss in East Antarctica within 200 years - Supplementary Materials

*James R. Jordan[a,b], B.W.J. Miles[c,d], G. H. Gudmundsson[a], S.S.R. Jamieson[c], A. Jenkins[a], C.R. Stokes[c]

[a]*Department of Geography and Environmental Sciences, Faculty of Engineering and Environment, Northumbria University, Newcastle upon Tyne, UK*
[b]*Laboratoire de Glaciologie, Université libre de Bruxelles (ULB), Brussels, Belgium*
[c]*Department of Geography, Durham University, Durham, DH1 3LE, UK*
[d]*School of Geosciences, University of Edinburgh, Edinburgh, UK*

Supplementary Methods

Ice Model

Mesh

The resolution of the initial ice model mesh is shown in Fig. S1. The mesh resolution varies from 250 m near the grounding line to 20 km further inland. Each year of simulated run time the mesh is further refined to track any migration of the grounding line

Calibration and validation

Úa is used to solve the equations of the shallow-ice stream or 'shelfy-stream' approximation (SSA[1]) rather than a shallow-ice sheet approximation. In accordance with the BedMachine data set[2] we have assumed a constant ice density of 917 kg m^{-3}. As direct observations of the basal slipperiness, C, and ice rate factor, A, are extremely limited and difficult to obtain we are forced to rely on indirect estimates, in this case via numerically inverting surface velocity measurements to obtain the corresponding values of A and C. The inversion process was performed for a range of values of m and n by minimizing the cost function of a misfit and regularization term. Úa uses the adjoint method to calculate the gradients of the cost function with respect to both A and C in a computationally efficient way. We impose regularization on A and C using Tikhonov regularization of both the amplitude and spatial

gradients of the A and C fields. We tested a series of regularization parameter values and selected final values based on an L-curve analysis. After 1000 iterations of the inversion process the mean difference between modelled and observed velocities was 6.2 m a^{-1}.

To test the impact of our assumed value for m upon ice volume above flotation, model inversions and simulations were carried out for m=1, m=2 and m=3. The results were found to be qualitatively similar, with a small variation in sensitivity to forcing of the order of several hundred Gt in VAF over the 200 year model simulation. Although there is a bias in our results imposed by our assumption of a value of m, as we are seeking to investigate the qualitative causes of future mass loss from East Antarctica rather than make exact predictions we can justify our use of a single value of m for our simulations. The results for the model inversion (using $m = 3$) performed to estimate the properties of East Antarctica that minimize the misfit between observed and modelled surface velocities are shown in Fig. S2. We use BedMachine bathymetry and ice thickness[2] along with MeASURES ice velocity for 2017[3]. As there is a slight temporal discrepancy in these two data sets, with BedMachine ice extent being composed from data from 1993–2016 and the MeASURES coverage being from 2017, we extend the ice shelf using nearest neighbour extrapolation of ice thickness. The observed (Fig. S2a) and modelled (Fig. S2b) ice velocity show a good level of agreement. Calculated values for the ice-rate factor, A, (Fig. S2c) show spatial variation, particularly in the faster flowing ice stream regions. Calculated values of basal slipperiness, C, (Fig. S2d), also have a high amount of spatial variation, with higher values indicating more slippery basal conditions. These areas of high basal slipperiness result in the high near-grounding line velocities seen in the observed (Fig. S2a) and modelled (Fig. S2b) ice velocities. Note that C is not directly calculated by the model inversion for the bed under floating ice, as until the ice is in contact with the bed the basal slipperiness has no impact upon ice velocity. To allow for more realistic bed properties if the grounding line were to advance past its initial position we impose nearest neighbour values of bed slipperiness under initially floating ice.

It should be noted that our choice of modelling framework limits the amount of physical processes accurately represented. Our model assumes ice shelves that have calving fronts that are constant in time and will never undergo a catastrophic breakup, such as occurred to Larsen B in 2002, although they are permitted to thin to zero ice thickness with a corresponding reduction in buttressing. We also assume that any surface melting caused

by the rising air temperature is frozen back into the ice sheet and as such do not account for the possibility of meltwater driven hydro fracturing of ice shelves. Despite this, we believe our results can offer valuable insight on the overall, qualitative trend of the EAIS mass balance even if individual ice shelves are misrepresented due to the lack of certain physical processes that may be important on a local level.

Ocean model

Calibration and validation

Accurately resolving CDW intrusions onto the continental shelf, and all the associated feedbacks in the ice-ocean systems, are problematic to numerically model due to needing a high-resolution, eddy-resolving ocean model that also simulates ice shelf cavities and melt water occurring at depth[4,5]. Direct simulations of CDW intrusions have been modelled by Morrison et al., 2020[6] showing the validity of the process, albeit with a model that does not fully resolve ice shelf cavities and with an ocean resolution higher than is commonly used in long term, CMIP style projections. Whilst the observational record is not extensive, recently published observations of oceanic conditions in East Antarctica by Herraiz-Borreguero and Garabato 2022[7] have observed a poleward shift in CDW in the period 2010-2018, suggesting that CDW intrusions in East Antarctica may become more likely in the future. Yamazaki et al., 2021[8] show evidence for a poleward shift of the southern boundary of the Antarctic Circumpolar Current over the last 30 years, leading to warming of onshelf waters. A study of the paleo record in the Wilkes Subglacial Basin by Crotti et al, 2022[9] found evidence linking grounding line retreat to periods of increased oceanic warming, most likely a result of CDW intrusions suggesting that they may occur more frequently in the future with a warming climate. Our approach for ocean forcing, therefore, is to simulate conditions that would occur from a process we know has occurred in the past and present and is likely to occur in the future, but that large scale ocean models lack the resolution to directly simulate. Our work focuses on the impact this would have on the EAIS mass balance, rather than concern ourselves with explicitly simulating the exact process by which this has occurred.

Ocean melt rates used to force the Úa ice model are determined by an implementation of the PICO (Potsdam Ice-shelf Cavity mOdel[10]), similar to that used by Hill et. al., 2021[11]. This uses a single representative ocean temperature and salinity, assumed to be located just outside the ice shelf

cavity, to derive its melt rate for an individual ice shelf. The melt rates are calculated for every single time step of the ice model, taking into account the changing geometry of ice shelf cavities. This process requires a certain minimal area of ice shelf to produce accurate results. For our model setup, it was determined that 4000 km^2 was a good minimum threshold (similar to to that used by Reese et al., 2018[12]), allowing us to model melt rates for 14 major East Antarctic ice shelves. Note that PICO counts any connected floating ice elements as part of the same ice shelf and, as such, several of these 14 ice shelves are actually a combination of 2 or more ice shelves. For instance, we treat Totten and Moscow University ice shelf as a combined ice shelf for purposes of model calibration. Any floating ice elements not part of these 14 individually modelled ice shelves are assigned the same melt rate, determined to be the area averaged model melt rate for the 14 explicitly modelled separate ice shelves, allowing it to also respond to changing oceanic forcings. In addition to ocean temperature, T_o, and salinity , S, PICO requires values for the strength of the overturning ocean circulation, C_p, and thermal heat exchange coefficient, γ_t. Present day oceanic conditions in the vicinity of our 14 East Antarctic ice shelves are derived from observations[13] Previous work by Reese et al., 2018[12] determined a physical range for values of C_p and γ_t for use with PICO. When calibrating each of the 14 ice shelves to match observed melt rates we preferentially tune C_p then γ_t and finally T_o and S. This is done on the basis that we consider C_p to be more likely to vary in respect to the ice shelf cavity geometry than γ_t whilst there are, in some cases, direct observations of T_o and S to inform the calibration process whilst directly observing C_p or γ_t in the field is a problematic issue. The results of the model calibration are shown in Fig. S3, with the initial properties used in this calibration shown in Table S1. A good agreement (within errors of observations) are obtained for all but Rennick and Jelbart ice shelf, with an overestimation of melt rates in both these cases.

Future oceanic forcing

Future oceanic conditions for both SW and mCDW under both the RCP4.5 and RCP 8.5 emissions scenarios are derived from an analysis of CMIP5 ensemble data investigating the average properties of Southern Ocean water masses under various emission scenarios[14]. We assume that at present all ice shelves are predominantly forced by SW. To represent a shift to being forced by mCDW we impose an additional change in T_o and S equal to the difference between the representative properties of SW and CDW in Sallée et. al.,

2013[14]. In reality we would expect mCDW intrusions to mix with ambient shelf water, lowering their temperature somewhat. Indeed, this is seen in CMIP6 ocean temperatures being less than our imposed mCDW forcing[15]. However, CDW intrusions have been shown to occur via submarine canyons[16] and CMIP models typically lack the resolution to fully resolve these features, potentially leading to an underestimation of CDW intruding onto the continental shelf. We justify our setup on the basis that it is designed to show an overall "worst case" scenario for future ocean warming due to a process we know occurs but currently have difficulty representing in large scale models.

The oceanic forcing conditions for the four climate scenarios are shown in Table S2. Examples of present day, calibrated oceanic melting as well as that after 200 years of the individual climate forcings scenarios are shown in Fig. S4. Throughout this work we have used CMIP5 ensemble data to derive our oceanic temperature forcings. However, a recent analysis of oceanic warming in CMIP6 models[15] shows increased warming in high emission scenarios when compared to CMIP5 models. This increased warming is still less than the warming brought about by a shift to modified Circumpolar Deep Water and thus should have a limited effect on our overall conclusions.

Atmospheric forcing

The RACMO version 2.3[17] is used to determine the spatial pattern of present day surface precipitation which is used to force the Úa ice model (Fig. S5). An evaluation of the best performing precipitation CMIP5 models by Palerme et, al., 2017[18] found that each degree of warming was equivalent to approximately a 7% increase in precipitation and that there was an increase of near surface air temperature of 2 degrees under the RCP4.5 scenario and 4 degrees under the RCP8.5 scenario. A slightly lower value of 5% ± 1% was obtained from ice core measurements, palaeo and future simulations be Frieler er al., 2015[19]. Note that this increase is compounded with each degree of warming, and so, for the sake of simplicity, we assume that this temperature increase is in the range of 4-7% and will occur linearly over the next 100 years, such that there is a 14% or 28% increase in surface precipitation by the end of this time period for the RCP4.5 and RCP8.5 emission scenarios, and scale the spatial pattern of present day precipitation by this percentage accordingly to account for future increases in precipitation. We assume that any surface melting arising from this increase in near surface air temperature will not result in significant melting, and any melt water that

does occur is refrozen into the ice sheet before leaving the model domain for the purpose of determining mass balance.

Reference run

Figure S6 shows the ice volume, VAF, floating ice and grounded area from the reference run over 20 years of simulation time with constant initial forcing. Whilst the reference run experiences minimal total ice volume change this is a result of a small increase in VAF being compensated by a small decrease in floating ice. Whilst there is an initial increase in grounded area over the first 50 years of the simulation, this is compensated by a fall in grounded area over the rest of the simulation period, resulting in a final grounded area close to the initial value.

Supplementary Discussion

Regional results

Figures S7–S14 show a closer look at the eight individual drainage basins covered by the model domain. In each case, volume above flotation, ice volume and grounded area are shown for the 200 years of model simulation relative to the baseline run. We also show mass balance and ice thickness change after the 200 years of simulated model time.

Acknowledgments

This work was funded by the Natural Environment Research Council, grant number NE/R000719/1. This publication was supported by PROTECT. James Jordan, Hilmar Gudmundsson and Adrian Jenkins have received funding from the European Union's Horizon 2020 research and innovation program under grant agreement No 869304, PROTECT contribution number 55. Bertie Miles was supported by a Leverhulme Early Career Fellowship (ECF-2021-484).

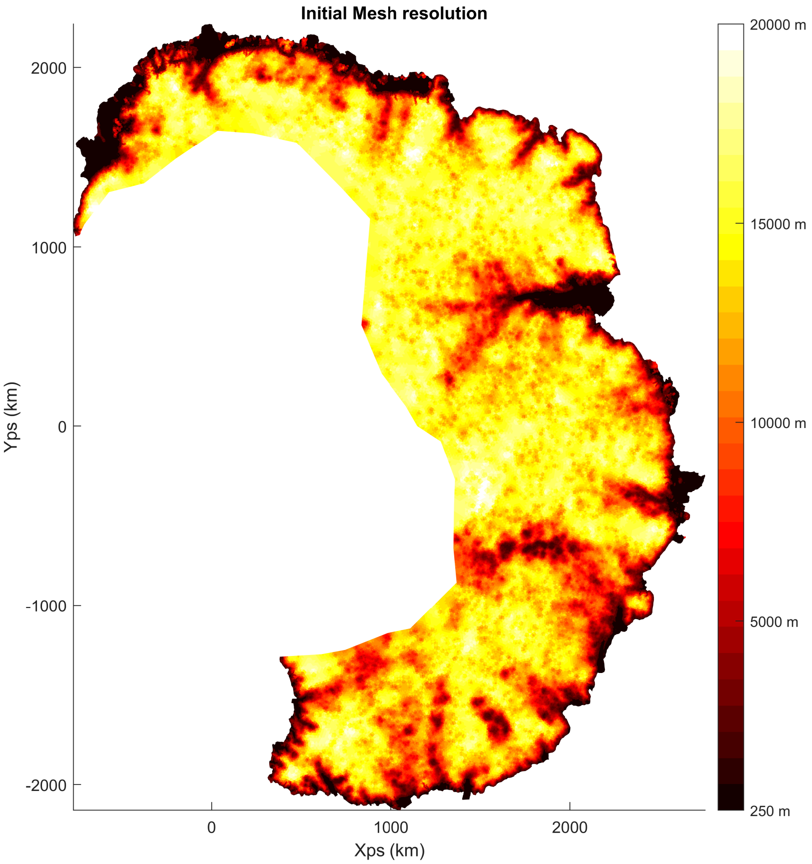

Figure S1: **Initial model mesh** Resolution varys from 20 km inland to 250m near the grounding line. Note that the model mesh is refined every year of the model simulations, with model elements within 5 km of the current grounding line position being given increased resolution.

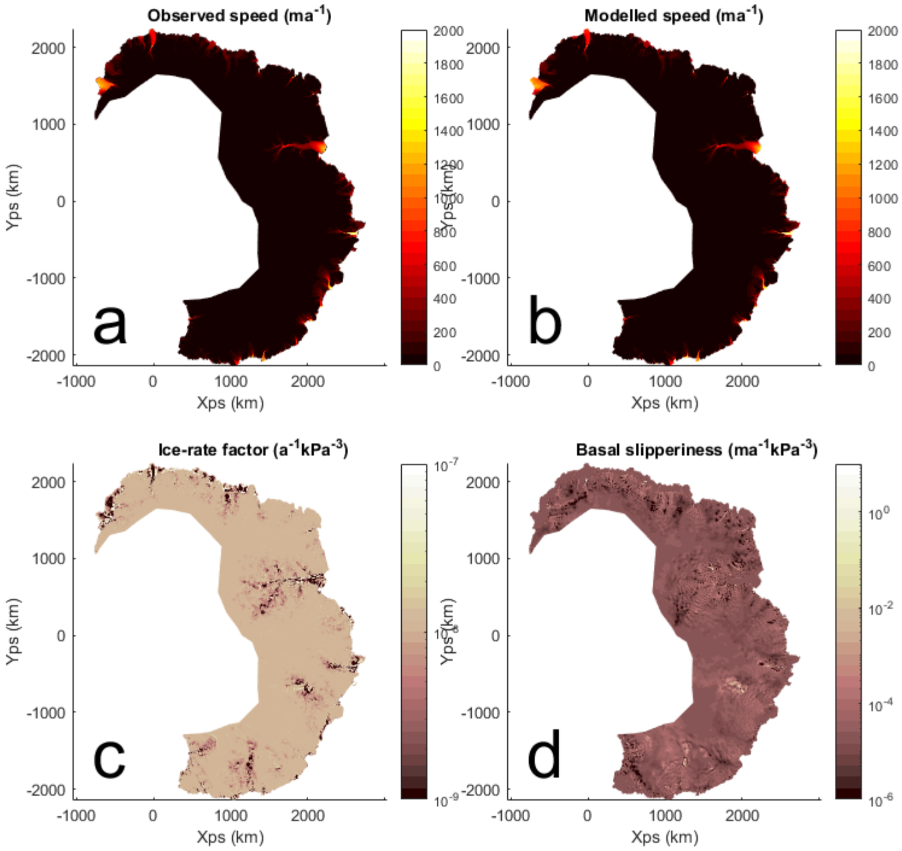

Figure S2: **Ice model inversion results.** (a) Observed MeASURES ice speed[3], (b) modelled ice speed using inverted properties plotted on model grid, (c) ice-rate factor, A, obtained from the inversion process and (d) basal slipperiness, C, obtained from the inversion process. Note that the standard Antarctic Polar Stereographic coordinates (EPSG:3031 WGS 84) are used.

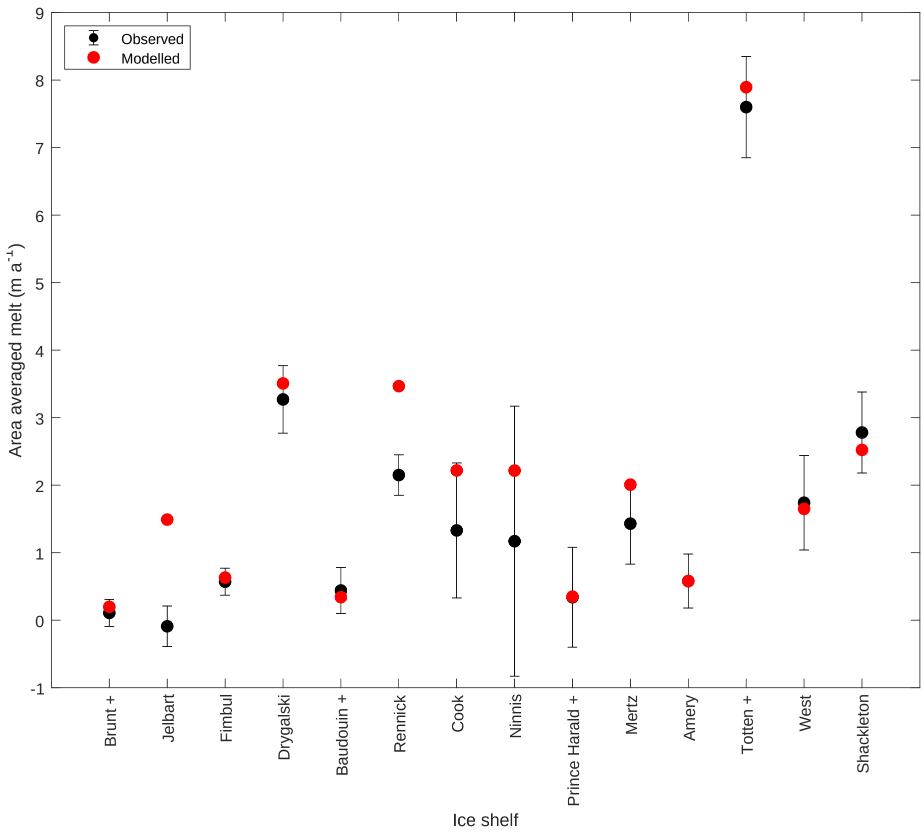

Figure S3: **Ocean box model calibration** Observed area averaged ice-shelf melt (Rignot et al., 2019[20], black with error bars) and initial modelled area averaged ice shelf (red) for the 14 major ice shelves in the model domain. Note that Brunt+ represents a weighted average of the Brunt, Stancomb and Riiser-Larsen ice shelves, and similarly with Baudouin+ (Baudouin, Borchgrevink and Lazarev), Prince Harald+ (Prince Harald and Shiraze) and Totten+ (Totten and Moscow University).

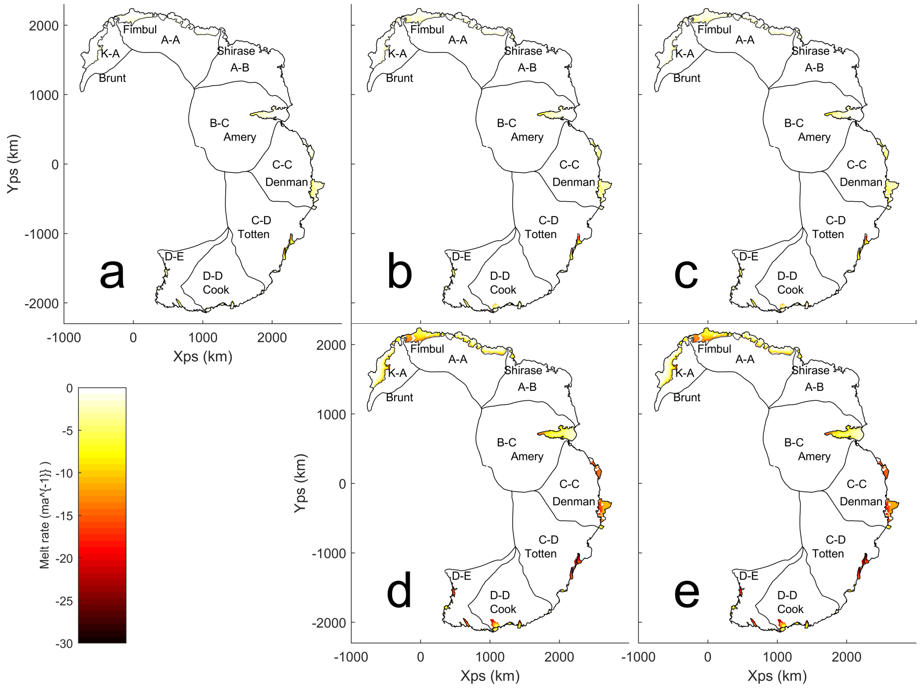

Figure S4: **Simulated melt rates** (a) calibrated present day and final modelled melt rates for (b) RCP4.5-SW, (c) RCP8.5-SW, (d) RCP4.5-mCDW and (e) RCP8.5-mCDW climate forcing scenario. (Representative Concentration Pathway, Shelf Water and Modified Circumpolar Deep Water respectively). Regions correspond to the IMBIE 2016 catchments[21]

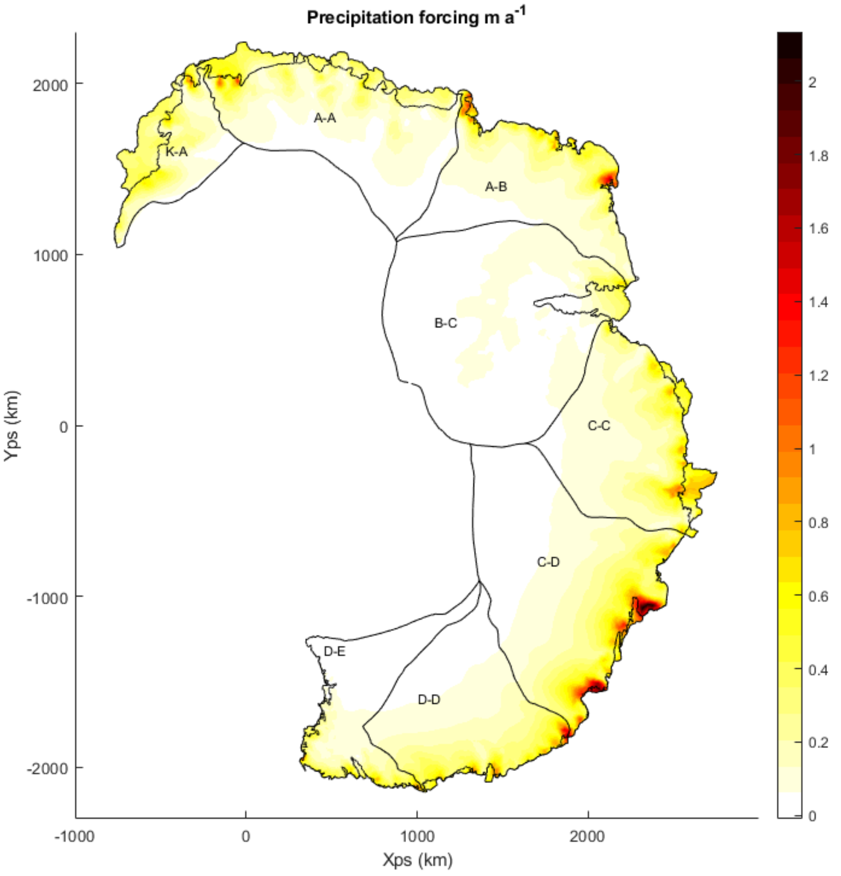

Figure S5: **Initial precipitation forcing.** Data is from the Regional Atmospheric Climate Model (RACMO) version 2.3 dataset[17]. Note, initial forcing is scaled by 7% per degree of atmospheric warming, i.e., by a maximum of 14% in RCP4.5 and 28% in RCP 8.5 scenarios (Representative Concentration Pathway) for the last 100 years of model simulations. Regions correspond to the IMBIE 2016 catchments[21]

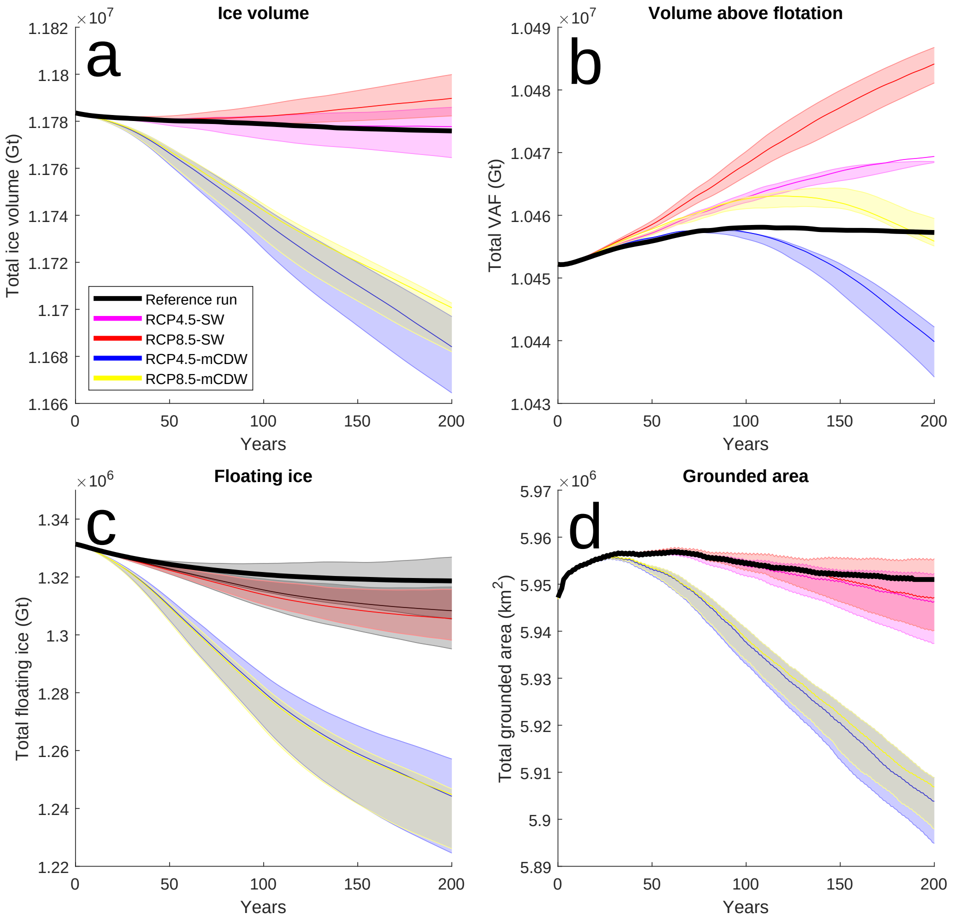

Figure S6: **Reference simulation results.** Ice volume (a), ice volume above flotation (b), floating ice volume (c), and grounded area (d) for the reference run (black). For comparative purposes the RCP4.5SW (magenta), RCP8.5-SW (red), RCP4.5-mCDW(blue) and RCP8.5-mCDW(yellow) emission scenarios are also shown (Representative Concentration Pathway, Shelf Water and Modified Circumpolar Deep Water respectively).

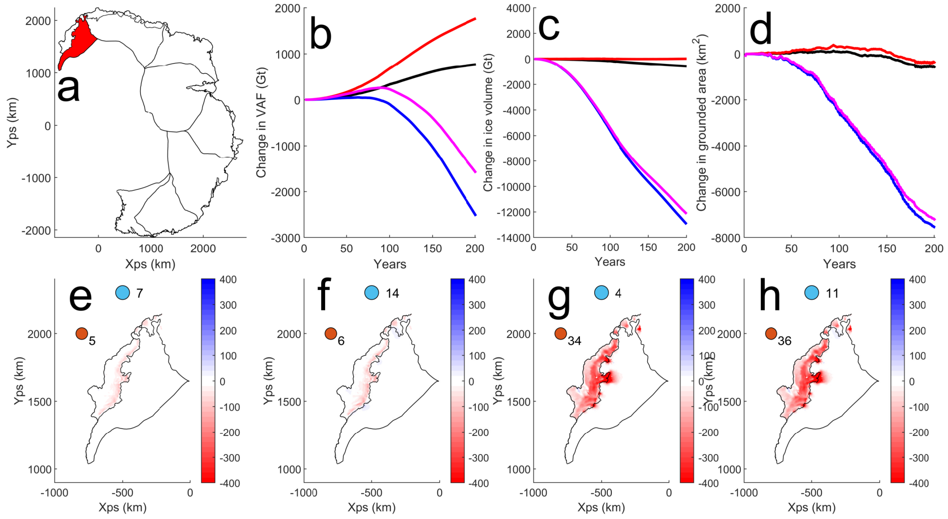

Figure S7: **Region K-A results.** (a) location of region K-A in relation to model domain, (b) Volume Above Flotation (VAF), (c) ice volume, and (d) grounded area from the four climate scenarios. Mass balance (red circle for ice discharge, blue circle for surface precipitation) and ice thickness change (colours) after 200 years simulation time relative to the baseline run for (e) RCP4.5-SW, (f) RCP8.5-SW, (g) RCP4.5-mCDW and (h) RCP8.5-mCDW climate scenarios (Representative Concentration Pathway, Shelf Water and Modified Circumpolar Deep Water respectively). Regions correspond to the IMBIE 2016 catchments[21]

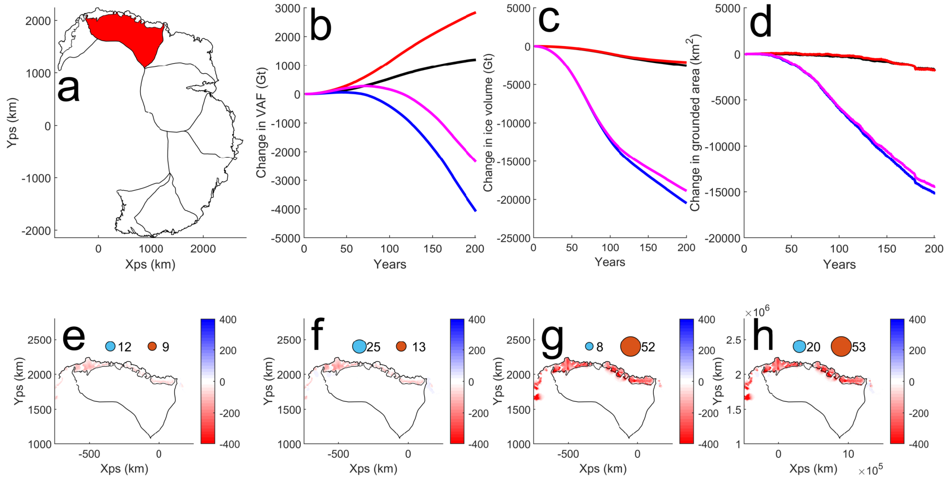

Figure S8: **Region A-A results.** (a) location of region K-A in relation to model domain, (b) Volume Above Flotation (VAF), (c) ice volume, and (d) grounded area from the four climate scenarios. Mass balance (red circle for ice discharge, blue circle for surface precipitation) and ice thickness change (colours) after 200 years simulation time relative to the baseline run for (e) RCP4.5-SW, (f) RCP8.5-SW, (g) RCP4.5-mCDW and (h) RCP8.5-mCDW climate scenarios (Representative Concentration Pathway, Shelf Water and Modified Circumpolar Deep Water respectively). Regions correspond to the IMBIE 2016 catchments[21]

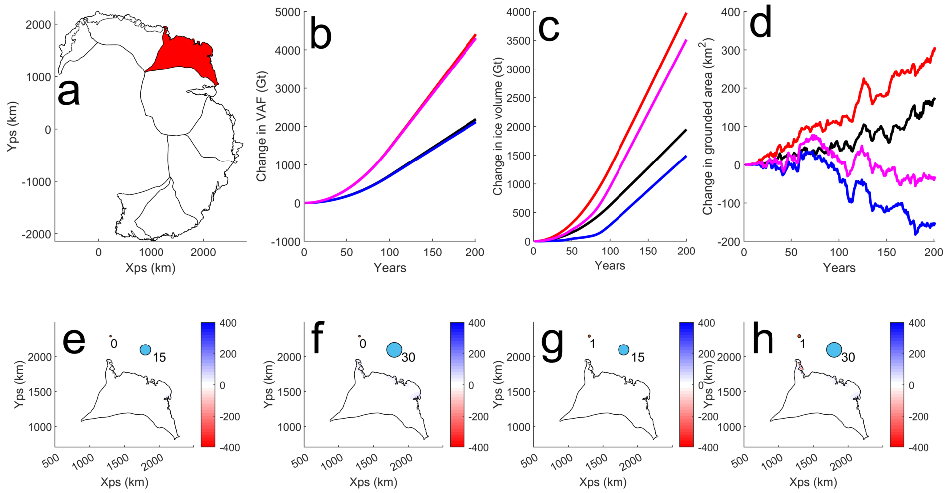

Figure S9: **Region A-B results.** (a) location of region K-A in relation to model domain, (b) Volume Above Flotation (VAF), (c) ice volume, and (d) grounded area from the four climate scenarios. Mass balance (red circle for ice discharge, blue circle for surface precipitation) and ice thickness change (colours) after 200 years simulation time relative to the baseline run for (e) RCP4.5-SW, (f) RCP8.5-SW, (g) RCP4.5-mCDW and (h) RCP8.5-mCDW climate scenarios (Representative Concentration Pathway, Shelf Water and Modified Circumpolar Deep Water respectively). Regions correspond to the IMBIE 2016 catchments[21]

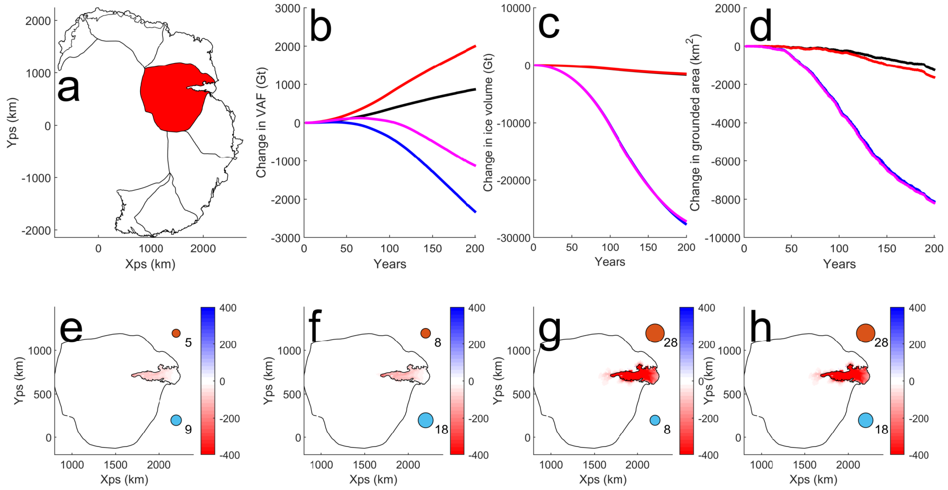

Figure S10: **Region B-C results.** (a) location of region K-A in relation to model domain, (b) Volume Above Flotation (VAF), (c) ice volume, and (d) grounded area from the four climate scenarios. Mass balance (red circle for ice discharge, blue circle for surface precipitation) and ice thickness change (colours) after 200 years simulation time relative to the baseline run for (e) RCP4.5-SW, (f) RCP8.5-SW, (g) RCP4.5-mCDW and (h) RCP8.5-mCDW climate scenarios (Representative Concentration Pathway, Shelf Water and Modified Circumpolar Deep Water respectively). Regions correspond to the IMBIE 2016 catchments[21]

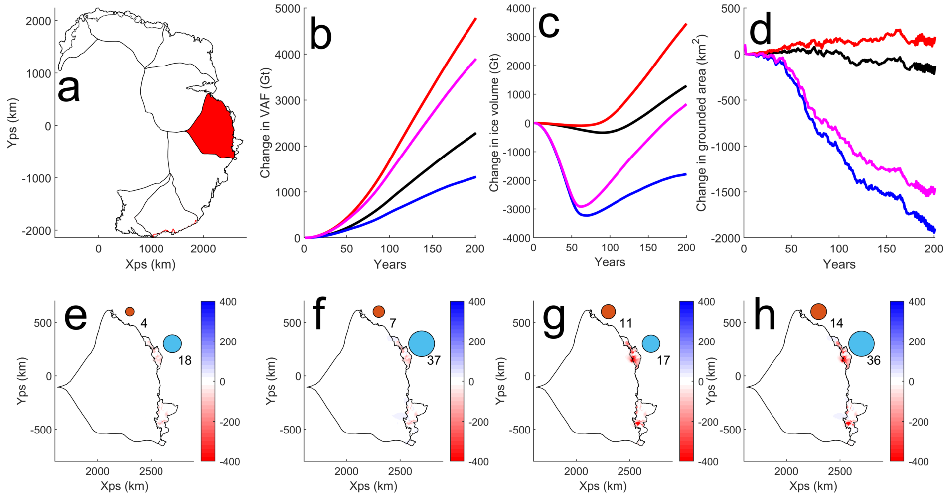

Figure S11: **Region C-C results.** (a) location of region K-A in relation to model domain, (b) Volume Above Flotation (VAF), (c) ice volume, and (d) grounded area from the four climate scenarios. Mass balance (red circle for ice discharge, blue circle for surface precipitation) and ice thickness change (colours) after 200 years simulation time relative to the baseline run for (e) RCP4.5-SW, (f) RCP8.5-SW, (g) RCP4.5-mCDW and (h) RCP8.5-mCDW climate scenarios (Representative Concentration Pathway, Shelf Water and Modified Circumpolar Deep Water respectively). Regions correspond to the IMBIE 2016 catchments[21]

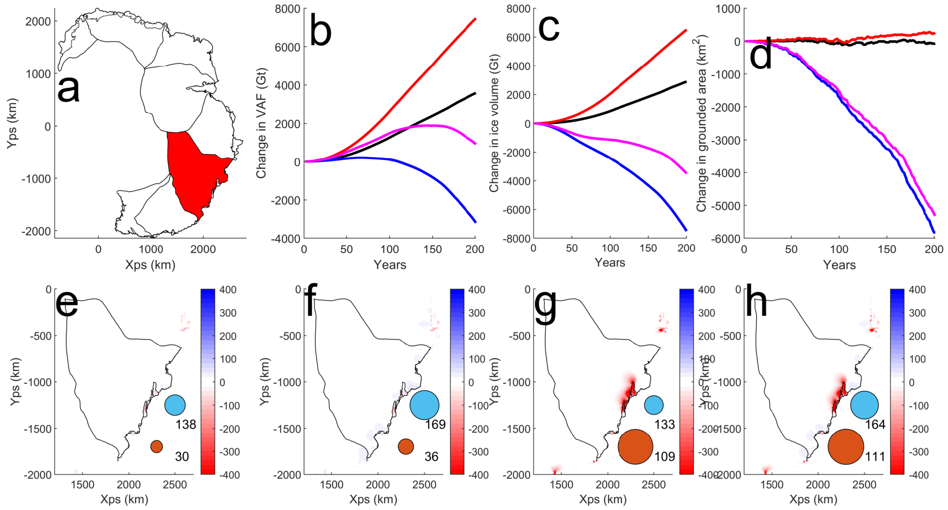

Figure S12: **Region C-D results.** (a) location of region K-A in relation to model domain, (b) Volume Above Flotation (VAF), (c) ice volume, and (d) grounded area from the four climate scenarios. Mass balance (red circle for ice discharge, blue circle for surface precipitation) and ice thickness change (colours) after 200 years simulation time relative to the baseline run for (e) RCP4.5-SW, (f) RCP8.5-SW, (g) RCP4.5-mCDW and (h) RCP8.5-mCDW climate scenarios (Representative Concentration Pathway, Shelf Water and Modified Circumpolar Deep Water respectively). Regions correspond to the IMBIE 2016 catchments[21]

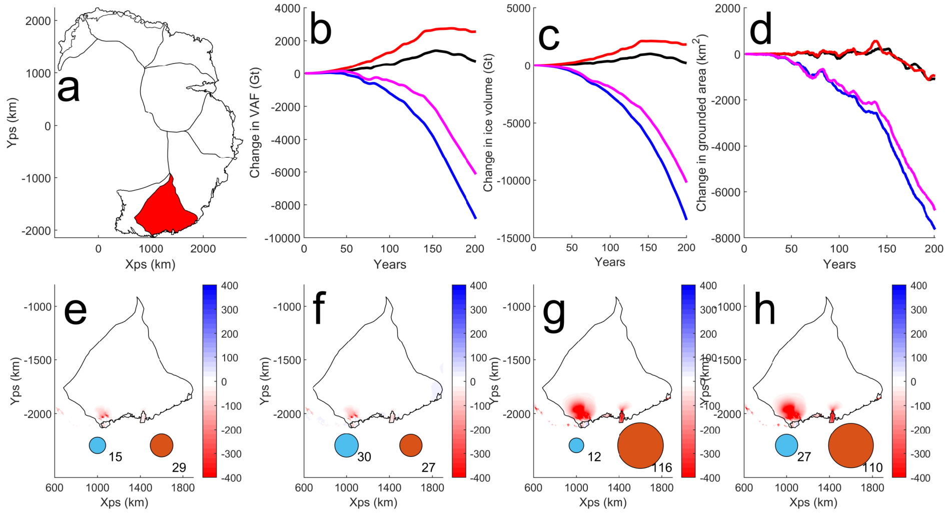

Figure S13: **Region D-D results.** (a) location of region K-A in relation to model domain, (b) Volume Above Flotation (VAF), (c) ice volume, and (d) grounded area from the four climate scenarios. Mass balance (red circle for ice discharge, blue circle for surface precipitation) and ice thickness change (colours) after 200 years simulation time relative to the baseline run for (e) RCP4.5-SW, (f) RCP8.5-SW, (g) RCP4.5-mCDW and (h) RCP8.5-mCDW climate scenarios (Representative Concentration Pathway, Shelf Water and Modified Circumpolar Deep Water respectively). Regions correspond to the IMBIE 2016 catchments[21]

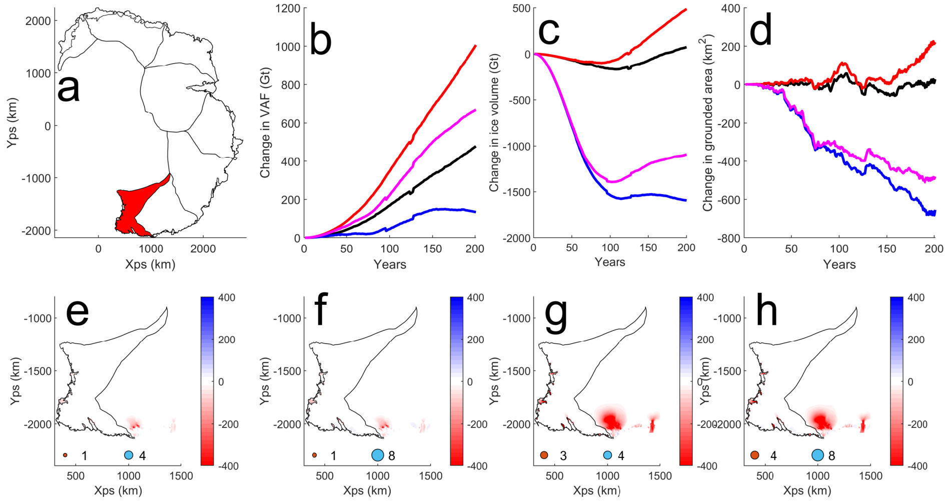

Figure S14: **Region D-E results.** (a) location of region K-A in relation to model domain, (b) Volume Above Flotation (VAF), (c) ice volume, and (d) grounded area from the four climate scenarios. Mass balance (red circle for ice discharge, blue circle for surface precipitation) and ice thickness change (colours) after 200 years simulation time relative to the baseline run for (e) RCP4.5-SW, (f) RCP8.5-SW, (g) RCP4.5-mCDW and (h) RCP8.5-mCDW climate scenarios (Representative Concentration Pathway, Shelf Water and Modified Circumpolar Deep Water respectively). Regions correspond to the IMBIE 2016 catchments[21]

Shelf Number	Name	T_o	S	C_p	g_t
1	Brunt+	-1.66	34.7	1e6	2e-5
2	Jelbart	-1.65	34.48	3e6	2e-5
3	Fimbul	-1.58	34.49	1e6	2e-5
4	Drygalski	-1.5	34.84	7e6	2e-5
5	Baudoin+	-1.3	34.84	0.6e6	3e-5
6	Rennick	-1.3	34.84	0.6e6	3e-5
7	Cook	-1.61	34.75	1e6	2e-5
8	Ninnis	-1.8	34.5	0.6e6	2e-5
9	Prince Harald	-1.61	34.75	0.7e6	2e-5
10	Mertz	-0.73	34.73	0.7e6	2e-5
11	Amery	-1.73	34.7	1e6	2e-5
12	Totten	-0.73	34.73	1e6	2e-5
13	West	-0.73	34.73	3e6	2e-5
14	Shackleton	-1.68	34.65	7e6	1e-5

Table S1: **Potsdam Ice-shelf Cavity mOdel (PICO) calibration parameters.** PICO calibration parameters for present day melt rates for 14 major East Antarctic ice shelves.

Climate scenario	T_o addition	S addition
RCP4.5-SW	0.262	0.013
RCP8.5-SW	0.356	0.023
RCP4.5-mCDW	1.925	-0.001
RCP8.5-mCDW	2.003	0.003

Table S2: **Climate scenario forcing.** Additional temperature and salinity forcing applied to the Potsdam Ice-shelf Cavity mOdel (PICO) calibration parameters to represent the four climate forcing scenarios (Representative Concentration Pathway, Shelf Water and Modified Circumpolar Deep Water respectively).

Supplementary References

[1] Kurt M Cuffey and William Stanley Bryce Paterson. *The physics of glaciers*. Academic Press, 2010.

[2] M. Morlighem, E. Rignot, and T. Binder. Deep glacial troughs and stabilizing ridges unveiled beneath the margins of the Antarctic Ice sheet. *Nature Geoscience*, 13:132–137, 2020.

[3] J. Mouginot, E. Rignot, and B. Scheuchl. Continent-wide, interferometric SAR phase, mapping of Antarctic ice velocity. *Geophysical Research Letters*, 46(16):9710–9718, 2019.

[4] Michael S. Dinniman, John M. Klinck, and Walker O. Smith. Cross-shelf exchange in a model of the Ross Sea circulation and biogeochemistry. *Deep Sea Research Part II: Topical Studies in Oceanography*, 50(22):3103–3120, 2003.

[5] Andrew L Stewart and Andrew F Thompson. Eddy-mediated transport of warm Circumpolar Deep water across the Antarctic shelf break. *Geophysical Research Letters*, 42(2):432–440, 2015.

[6] AK Morrison, A McC Hogg, Matthew Heathcote England, and P Spence. Warm Circumpolar Deep Water transport toward Antarctica driven by local dense water export in canyons. *Science Advances*, 6(18):eaav2516, 2020.

[7] Laura Herraiz-Borreguero and Alberto C Naveira Garabato. Poleward shift of Circumpolar Deep Water threatens the East Antarctic Ice Sheet. *Nature Climate Change*, pages 1–7, 2022.

[8] Kaihe Yamazaki, Shigeru Aoki, Katsuro Katsumata, Daisuke Hirano, and Yoshihiro Nakayama. Multidecadal poleward shift of the southern boundary of the Antarctic Circumpolar Current off East Antarctica. *Science Advances*, 7(24):eabf8755, 2021.

[9] Ilaria Crotti, Aurélien Quiquet, Amaelle Landais, Barbara Stenni, David J Wilson, Mirko Severi, Robert Mulvaney, Frank Wilhelms, Carlo Barbante, and Massimo Frezzotti. Wilkes subglacial basin ice sheet response to Southern Ocean warming during late Pleistocene interglacials. *Nature Communications*, 13(1):1–11, 2022.

[10] R. Reese, T. Albrecht, M. Mengel, X. Asay-Davis, and R. Winkelmann. Antarctic sub-shelf melt rates via PICO. *The Cryosphere*, 12(6):1969–1985, 2018.

[11] E. A. Hill, S. H. R. Rosier, G. H. Gudmundsson, and M. Collins. Quantifying the potential future contribution to global mean sea level from the Filchner–Ronne basin, antarctica. *The Cryosphere*, 15(10):4675–4702, 2021.

[12] R. Reese, G. H. Gudmundsson, A. Levermann, and R. Winkelmann. The far reach of ice-shelf thinning in Antarctica. *Nature Climate Change*, 8:53–57, 2018.

[13] S. Schmidtko, K. Heywood, A. Thompson, and S. Aoki. Multidecadal warming of Antarctic waters. *Science*, 346:1227–1231, 2014.

[14] J.-B. Sallée, E. Shuckburgh, N. Bruneau, A. J. S. Meijers, T. J. Bracegirdle, Z. Wang, and T. Roy. Assessment of Southern Ocean water mass circulation and characteristics in CMIP5 models: Historical bias and forcing response. *Journal of Geophysical Research: Oceans*, 118(4):1830–1844, 2013.

[15] Ariaan Purich and Matthew H. England. Historical and future projected warming of Antarctic Shelf Bottom Water in CMIP6 models. *Geophysical Research Letters*, 48(10):e2021GL092752, 2021.

[16] Giorgio Budillon, Pasquale Castagno, Stefano Aliani, Giancarlo Spezie, and Laurie Padman. Thermohaline variability and Antarctic bottom water formation at the Ross Sea shelf break. *Deep Sea Research Part I: Oceanographic Research Papers*, 58(10):1002–1018, 2011.

[17] J.M. Van Wessem, C.H. Reijmer, M. Morlighem, J. Mouginot, E. Rignot, B. Medley, I. Joughin, B. Wouters, M.A. Depoorter, and J.L Bamber. Improved representation of East Antarctic surface mass balance in a regional atmospheric climate model. *Journal of Glaciology*, 60(222):761–770, 2014.

[18] C. Palerme, C. Genthon, C. Claud, J. Kay, N. Wood, and T. L'Ecuyer. Evaluation of current and projected Antarctic precipitation in CMIP5 models. *Climate Dynamics*, 48:225–239, 2017.

[19] Katja Frieler, Peter U Clark, Feng He, Christo Buizert, Ronja Reese, Stefan RM Ligtenberg, Michiel R Van Den Broeke, Ricarda Winkelmann, and Anders Levermann. Consistent evidence of increasing Antarctic accumulation with warming. *Nature Climate Change*, 5(4):348–352, 2015.

[20] E. Rignot, S. Jacobs, J. Mouginot, and B. Scheuchl. Ice-shelf melting around Antarctica. *Science*, 341(6143):266–270, 2013.

[21] H. Jay Zwally, Mario B. Giovinetto amd Matthew A. Beckley, and Jack L. Saba. Antarctic and Greenland drainage system, 2012.
